# Investigations into the effects of scaffold microstructure on slow-release system with bioactive factors for bone repair

**DOI:** 10.3389/fbioe.2023.1230682

**Published:** 2023-09-14

**Authors:** Baoqing Pei, Mengyuan Hu, Xueqing Wu, Da Lu, Shijia Zhang, Le Zhang, Shuqin Wu

**Affiliations:** ^1^ Beijing Key Laboratory for Design and Evaluation Technology of Advanced Implantable and Interventional Medical Devices, Beijing Advanced Innovation Center for Biomedical Engineering, School of Biological Science and Medical Engineering, Beihang University, Beijing, China; ^2^ School of Big Data and Information, Shanxi College of Technology, Taiyuan, Shanxi, China

**Keywords:** bone tissue engineering, bioactive factor, slow-release system, scaffold microstructure, bone repair

## Abstract

In recent years, bone tissue engineering (BTE) has played an essential role in the repair of bone tissue defects. Although bioactive factors as one component of BTE have great potential to effectively promote cell differentiation and bone regeneration, they are usually not used alone due to their short effective half-lives, high concentrations, *etc.* The release rate of bioactive factors could be controlled by loading them into scaffolds, and the scaffold microstructure has been shown to significantly influence release rates of bioactive factors. Therefore, this review attempted to investigate how the scaffold microstructure affected the release rate of bioactive factors, in which the variables included pore size, pore shape and porosity. The loading nature and the releasing mechanism of bioactive factors were also summarized. The main conclusions were achieved as follows: i) The pore shapes in the scaffold may have had no apparent effect on the release of bioactive factors but significantly affected mechanical properties of the scaffolds; ii) The pore size of about 400 μm in the scaffold may be more conducive to controlling the release of bioactive factors to promote bone formation; iii) The porosity of scaffolds may be positively correlated with the release rate, and the porosity of 70%–80% may be better to control the release rate. This review indicates that a slow-release system with proper scaffold microstructure control could be a tremendous inspiration for developing new treatment strategies for bone disease. It is anticipated to eventually be developed into clinical applications to tackle treatment-related issues effectively.

## 1 Introduction

Bone defects are common diseases caused by infection, trauma, or congenital physical problems. In the case of mild bone damage, bone defects can be cured by the regenerative ability of bone tissue. But when the bone defects exceed the critical-size defects (CSDs), bone transplants are usually required, which include autografts, allografts, and synthetic biomaterials (Bishop and Einhorn, 2007; García-Gareta et al., 2015; Tozzi et al., 2016; El-Rashidy et al., 2017; Xu et al., 2017). Autografts have been considered the “gold standard” of bone treatment due to their excellent biocompatibility, osteoinduction, and exemption of immune responses. Nevertheless, autografts might lead to some complications because of the secondary surgery (Myeroff and Archdeacon, 2011; Amini et al., 2012). Allografts are widely accessible, but are subject to immunological rejection that requires a long time to resolve. Additionally, synthetic biomaterials are readily available, but they might be corroded in the body, leading to toxic phenomena (Ding et al., 2016; Chu et al., 2018). A brand-new field of research called BTE appears to be an effective way to treat bone defects (El-Rashidy et al., 2017).

**FIGURE 1:**
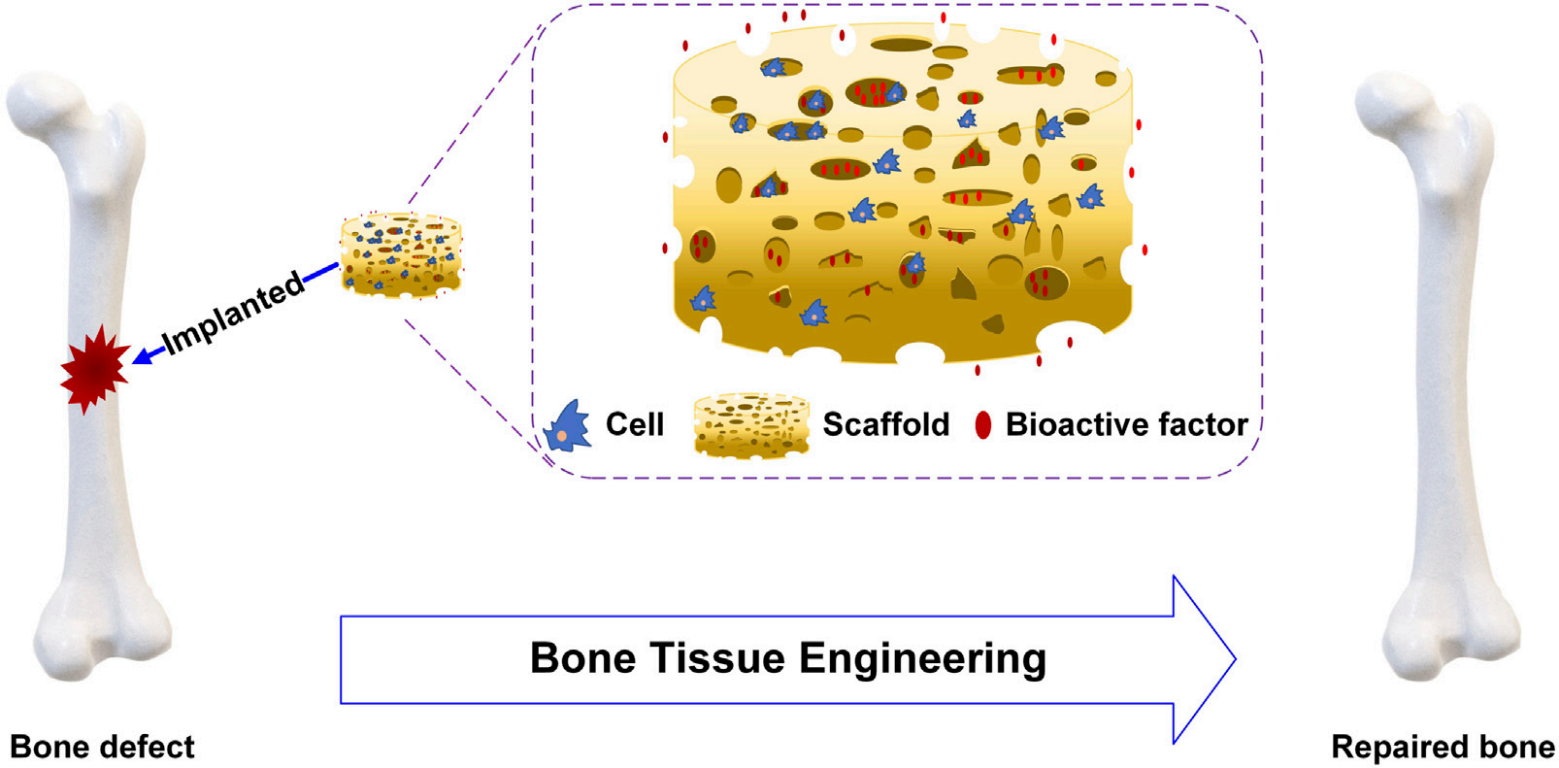
Schematic diagram of bone tissue engineering.

BTE constructs tissues or organs *in vitro* or *in vivo*, combining cell biology and biomaterials. For BTE, scaffolds, cells and bioactive factors are the three important elements to promote bone repair and regeneration, as shown in Figure 1. The ideal scaffolds should be biocompatible and biodegradable, have superior mechanical properties similar to those of bone transplant sites, and provide a stable three-dimensional environment to enhance cell adhesion and proliferation (Ratner et al., 2004; Meyers et al., 2008; Khaled et al., 2011). Stem cells are characterized by their ability to renew themselves and differentiate into various cell types. The presence of undifferentiated stem cells, which replace damaged differentiated cells, is essential for the success of osteoinduction (Heydarkhan-Hagvall et al., 2012; Palm et al., 2013). Bioactive factors are mostly peptides such as bone morphogenetic proteins (BMPs) which can bind to cell membrane receptors to enhance cell proliferation and differentiation (Castro et al., 2015; Ding et al., 2016; Ghorbani et al., 2021).

However, there are some limitations on bioactive factors: i) They are water-soluble signaling molecules, which cannot exist stably in aqueous solution; ii) It is challenging to maintain bioactivities over an extended period since the intrinsic half-life is short in the physiological environment; iii) The inappropriate concentration could bring various degrees of effects. The sudden release resulted in the localized high-concentration killing of cells, and the ossification was not evident at low concentrations (Srouji et al., 2011; Bhakta et al., 2012; Vo et al., 2012; Perez et al., 2014). In response to these shortcomings, a slow-release system composed of scaffolds and bioactive factors should be produced to precisely release bioactive factors to the sites of local defects and control the release sequence and release rate (Vo et al., 2012; Perez et al., 2014). By controlling the release of bioactive factors, the slow-release system could stimulate cell differentiation, enhance blood vessel formation and promote bone repair efficiently. Schliephake et al. (Schliephake et al., 2007) investigated a slow-release system of polylactic acid and bone morphogenetic protein-2 (rhBMP-2). They indicated that the slow release was possible, and the system had significant osteoinduction. Additionally, modification of the scaffold microstructure could alter the release characteristics to influence the performance of slow-release systems for bone repair.

To comprehend how the scaffold microstructure affected a slow-release system, we expounded and summarized the requirements of ideal scaffolds, preparation methods of the scaffolds, and especially some characteristics of scaffold microstructure. Next, we outlined the incorporation strategies between scaffolds and bioactive factors and the releasing mechanism of bioactive factors. Finally, we primarily elucidated the impacts of scaffold microstructure with pore shape, pore size and porosity on the release of bioactive factors.

## 2 Bone tissue engineering scaffolds

As is well known, bioactive factors need to reach the damaged sites with the appropriate bioactivity within a certain time to stimulate bone regeneration. However, this process can be unsuccessfully due to the disadvantages of bioactive factors. A legitimate way to reduce these defects is by incorporating bioactive factors into scaffolds to form a slow-release system. Furthermore, the scaffold microstructure could be modified to obtain the ideal controlled-release performance (Vo et al., 2012). The scaffold carriers should meet the following requirements (Vo et al., 2012; Arafat et al., 2014): i) superior biocompatibility and mechanical properties without physical damage; ii) appropriate biodegradability so that the scaffolds degrade with the process of bone regeneration; iii) delivery of bioactive factors to the site of the defects for controlled release.

The scaffold biomaterials include four types (Sehgal et al., 2017; Weißmann et al., 2017; Zhao S. et al., 2018; Zaharin et al., 2018; El-Habashy et al., 2021): i) biomedical metal materials (Weißmann et al., 2017; Zhao S. et al., 2018; Zaharin et al., 2018) such as stainless steel and titanium alloy; ii) bioceramics like bioactive glass, hydroxyapatite and tricalcium phosphate; iii) polymers including natural polymers like silk fibroin and chitosan, and synthetic polymers like polylactic acid, polycaprolactone and tricalcium phosphate; iv) composites such as polylactic acid-hydroxyapatite. Scaffolds are developed by combining one or more biomaterials, including nano-biomaterials, to achieve the desired performance (Al-Munajjed et al., 2008; Sicchieri et al., 2012; Zhao H. et al., 2018; Zaharin et al., 2018; Lee et al., 2019). Scaffolds are manufactured by using conventional techniques such as solvent casting, electrospinning and freeze-drying (Ferrand et al., 2014; Weißmann et al., 2017), and 3D printing technologies like stereolithography, selective laser melting and fused deposition modeling (Zhao H. et al., 2018; Lee et al., 2019). A summary of scaffold materials and techniques for BTE is provided in Table 1.

**TABLE 1:** Summary of materials and techniques for BTE scaffolds.

Scaffold materials	Examples	Technique	Features	References
Metals	Titanium alloy magnesium alloy tantalum	3D printing plasma spraying	High young’s modulus high compressive strength	Wauthle et al. (2015); Agarwal et al. (2016); Peng et al. (2016); Liu et al. (2017); Tamaddon et al. (2017); Zaharin et al. (2018)
		sintered metal powders	Low biocompatibility non-degradability	
		electron beam melting	corrosivity	
Bioceramics	Bioglass silicate alumina	3D printing co-precipitation method	Excellent biocompatibility convenient osteoinductivity	Sha et al. (2011); Eckel et al. (2016); Elsayed et al. (2017); Tang et al. (2018), Fu et al. (2019); Zhao et al. (2020)
		selective laser sintering	High brittleness poor mechanical properties	
Natural polymers	Collagen silk fibroin	3D printing electrospinning	Excellent biocompatibility and osteoinductivity low immune response	Castro et al. (2015); Loessner et al. (2016); Lee et al. (2019); Luo et al. (2021b)
	chitosan		Poor mechanical properties	
	hyaluronic acid			
Synthetic polymers	Polylactic acid polyglycolic acid	3D printing electrospinning	Good biocompatibility and degradability high mechanical strength	Aragon et al. (2017); Sruthi et al. (2020); Awasthi et al. (2021); Rachmiel et al. (2021)
	polycaprolactone		Fast degradation of some materials inflammatory response	
Composites	Polylactic-co-glycolic acid silk-hydroxyapatite hydroxyapatite-polycaprolactone	3D printing freeze drying	Excellent comprehensive performance wide range of materials	Ding et al. (2016); Xu et al. (2017); Zhou et al. (2017); Marins et al. (2019); Chen et al. (2020); El-Habashy et al. (2021)
		chemical precipitation	Complicated preparation process	
		electrospinning		

Some scaffolds are designed to mimic bone tissue’s mechanical properties and microstructures (Wu et al., 2020). Researchers have fabricated a variety of scaffolds to investigate the impact caused by microstructures like pore shape, pore size and porosity (Moroni et al., 2006; Van Bael et al., 2012; Liu et al., 2013; Prochor and Gryko, 2020; Li et al., 2021). Among them, Bael et al. (Van Bael et al., 2012) designed six Ti6Al4V scaffolds with three distinct pore shapes (triangular, hexagonal and rectangular) and pore sizes of 500 μm and 1,000 μm. The research indicated that the differentiation of human periosteum-derived cells (hPDC) was reliant on both pore shapes and pore sizes, and hPDC proliferation was related to pore sizes. Analogously, Prochor and Gryko (2020) investigated scaffolds with five different pore shapes to evaluate the effect on osteogenic cell diffusion. There have been numerous studies done on the pore sizes of scaffolds (Liu et al., 2013; Zhao et al., 2017; De Witte et al., 2018; Diao et al., 2018; Zaharin et al., 2018; Luo C. et al., 2021; Ghorbani et al., 2021; Sun et al., 2021). Luo et al. (Luo C. et al., 2021) demonstrated that porous tantalum scaffolds with pore sizes of 400–600 μm were more appropriate to promote cell adhesion, cell proliferation and bone regeneration compared to the other porous tantalum scaffolds with pore sizes of 100–200, 200–400, and 600–800 μm. Another critical component of the scaffold microstructure is porosity (Peng et al., 2012; Wo et al., 2020; Mohammadi et al., 2021; Foroughi and Razavi, 2022; Isaacson et al., 2022; Jeyachandran et al., 2022; Wang et al., 2022). Isaacson (Isaacson et al., 2022) examined hydroxyapatite gyroid scaffolds with 60%, 70% and 80% porosities. They considered the mechanical properties of scaffolds with 60% and 70% porosities comparable to those of cancellous bone.

The scaffold was a significant component of a slow-release system. Interconnected pores were more conducive to bone regeneration, especially when the pore size for angiogenesis was greater than 50 μm (Huang et al., 2022). Previous studies have shown that the pore shapes impacted the characteristics of cells, and scaffolds with elliptic pores had tremendous potential for bone regeneration (Boccaccio et al., 2016). Hence, developing scaffolds with balanced performance is equally challenging.

## 3 The mechanism of bioactive factors on slow-release system

Bone repair encompasses three categories of bioactive factors: inflammatory factors (such as fibroblast growth factors (FGF-II), transforming growth factor-β1 (TGF-β1), vascular endothelial growth factor (VEGF)), angiogenic factors (such as platelet-derived growth factor (PDGF) and VEGF), and osteogenic factors (such as BMPs and FGF). These bioactive factors were incorporated into scaffolds using various strategies to achieve specific release effects. Understanding bioactive factors’ releasing mechanism was crucial to the design of sustained delivery systems. This section predominantly addressed the loading nature and releasing mechanisms of bioactive factors.

### 3.1 The loading nature of bioactive factors

**FIGURE 2:**
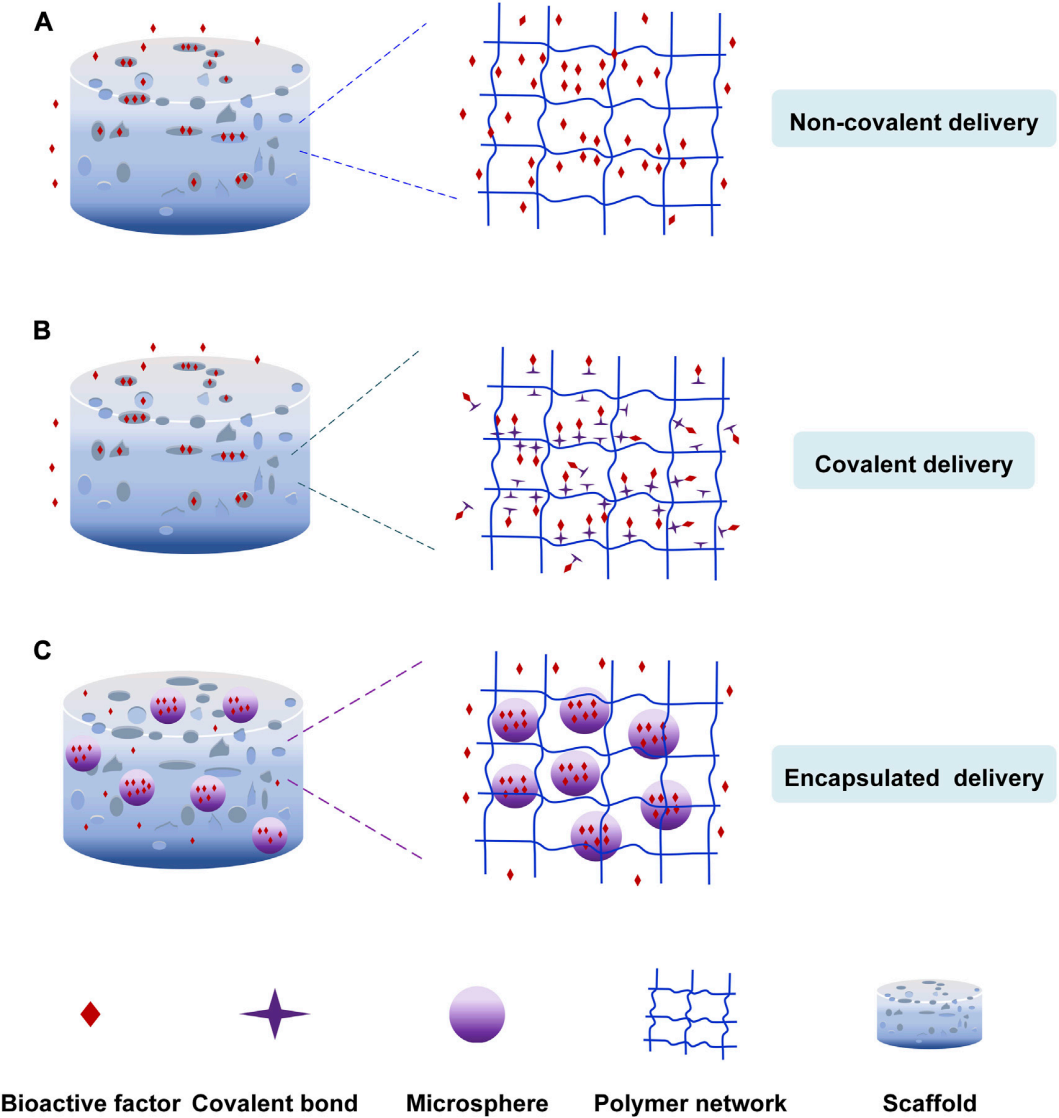
The loading nature of bioactive factors. **(A)** Non-covalent delivery. **(B)** Covalent delivery. **(C)** Encapsulated delivery.

The release of bioactive factors can be controlled by incorporating them into scaffolds through non-covalent bonding, covalent bonding and particulate encapsulation (Figure 2). Non-covalent bonding is achieved via physical adsorption, affinity interaction, electrostatic interaction, hydrogen bonding, *etc.* (Ziegler et al., 2008; Chen et al., 2009). Covalent bonding is achieved by coupled chemical reactions with scaffold biomaterials (Schliephake, 2010; Bouyer et al., 2016). In addition, the factors were loaded into scaffolds after they had been encapsulated in nanoparticles or microspheres to control the sequence and concentration of release of growth factors (Ehrbar et al., 2007; Balasubramanian et al., 2010; Bhakta et al., 2012).

#### 3.1.1 Non-covalent immobilization of bioactive factors

Physical adsorption is the simplest non-covalent bonding method, in which scaffolds are immersed in the bioactive factor solution to diffuse bioactive factors into scaffolds (Ziegler et al., 2008; Chen et al., 2009). For example, Sundaray et al. (Sundaray et al., 2011) prepared the nanofibrous scaffold loading the nerve growth factor through physical adsorption to direct the growth of neurites. Mehnath et al. (Mehnath et al., 2021) produced the mesoporous bioactive glass (MBG) scaffold to treat bone metastasis cancer. The hyaluronic acid-alendronate (HA-ALN) components (bone-targeting components) enclosed the MBG nanoparticles to form a stable structure to control the drug escape. The doxorubicin (DOX) was adsorbed into the nanoparticles via Van Der Waals force, which the pores can keep the sustained release of DOX. Lee et al. (Lee et al., 2012) prepared the heparin-conjugated electrospun poly (ε-caprolactone) (PCL)/gelatin scaffolds loading platelet-derived growth factor-BB (PDGF-BB). They discovered that the non-heparinized scaffold led to the burst release of PDGF-BB because of the physical adsorption. But the heparin-conjugated scaffold had the sustained release of PDGF-BB. Bioactive factors could also be incorporated into scaffolds through intermolecular hydrogen bonding, affinity interaction, *etc.* Song et al. (Song et al., 2021) developed a nanofibrous composite-coated titanium implant via electrospinning to enhance bone regeneration. They prepared the electrospun fiber composites using minerals (Zn, Mg, Si) substituted hydroxyapatite (MHAP), Polyethylene Glycol (PEG)/Cissus quadrangularis (CQ) extract. Cissus quadrangularis can be used to treat various ailments such as catagma, osteoarthritis, *etc.* CQ extract and polymers were bonded by hydrogen bonding, and they could promote cell differentiation.

Due to their independence from scaffolds, the bioactive factors have higher bioactivity and are released by diffusion and scaffold degradation (Figure 2A). The release of bioactive factors is influenced by pore size, porosity, interconnectivity, *etc.* (Izadifar et al., 2014). For example, Yang et al. (Yang et al., 2023) developed a hydrogel-based scaffold combined with nanofibrous. The 3D scaffold with porous architecture and interconnected pores accelerated osteanagenesis, cell proliferation, and controlled chemical release. When the pore size of the scaffold is larger than the size of the growth factor, the growth factor could spontaneously diffuse. Otherwise, the bioactive factors diffused after the degradation of scaffolds (Censi et al., 2012). The limitations are the low loading rates and the abrupt release of bioactive factors in a preliminary period. Surface roughness, wettability, and microstructure of scaffolds can control the release and improve the loading rates of bioactive factors.

#### 3.1.2 Covalent immobilization of bioactive factors

The second delivery strategy is incorporating bioactive factors into scaffolds in a covalent method (Figure 2B) (Kashiwagi et al., 2009; Kang et al., 2010; Schliephake, 2010; Tsurushima et al., 2010; Ehlert et al., 2011). The bioactive factors are incorporated into scaffolds covalently, or the bioactive factors are covalently loaded onto the modified scaffold surfaces via functional groups like primary amine, carboxyl, *etc.* The chemical binding between bioactive factors and scaffolds influenced the release of factors. The covalent delivery systems are more stable and have a longer extended-release time than non-covalent delivery systems owing to the covalent loading. Capsaicin (CAP) could kill cancer cells and promote cell proliferation. Murugan et al. (Murugan et al., 2018) loaded the capsaicin into the HAP/PXS scaffold (nano-hydroxyapatite with poly (xylitol sebacate) PXS co-polymer) to deal with osteosarcoma disease. The capsaicin was incorporated with HAP/PXS composite via intra-molecular forces of the hydroxyl group to keep the sustained release to develop bone regeneration. Prabakaran et al. (Prabakaran et al., 2021) prepared the substituted hydroxyapatite-starch-clay bio-composite and deposited it on the Ti plate. The ability of cell differentiation and Ca mineralization was more impressive due to the presence of Mg^2+^ and Gd^3+^. The research carried out by Arjama et al. (Arjama et al., 2021) demonstrates that the biomaterial of silk fibroin conjugated hyaluronic acid-hydroxyapatite hydrogel can be considered as the natural hydrogel to improve osteogenesis. The hydrogel is beneficial to entrap bioactive factors because of its exceptional mechanical properties. The anticancer drug DOX was covalently linked to polyphosphazene polymer to form the stable structure, and an amide bond was created through a chemical reaction between the secondary amine of DOX and carboxylic acid groups of polymers. The system released DOX sustainedly and controllably to inhibit the growth of bone cancer cells (Chun et al., 2009). Ham et al. (Ham et al., 2017) found a method for site-specific covalent immobilization. They immobilized azide-tagged engineered interferon-γ to control the differentiation of neural stem cells. The covalent delivery strategy improves the loading efficiency and reduces the burst release. But, the chemical binding and the mechanism of hydrolysis or enzymolysis might impair the biological activity of bioactive factors, leading to a bioactive decline. (Cross et al., 2003; Kashiwagi et al., 2009; Schliephake, 2010; Vo et al., 2012).

#### 3.1.3 Encapsulation of bioactive factors

The particulate encapsulation is the third delivery strategy that bioactive factors are encapsulated into nanoparticles or microspheres made of biodegradable materials like gelatin and chitosan through gas foaming, solvent casting and freeze-drying, *etc.* (Meinel et al., 2003; Wenk et al., 2009; Lee et al., 2011) and then they are incorporated into scaffolds to repair the area of the bone defect (Figure 2C) (Chu et al., 2007; Kashiwagi et al., 2009). The release mechanism of this loading strategy involves diffusion of bioactive factors, degradation of nanoparticles or microspheres, and scaffold degradation (Lee et al., 2011; Vo et al., 2012). Zhao et al. (Zhao et al., 2021) formulated a carbon nanotube-reinforced hydroxyapatite scaffold loading gold nanoparticles. The hydroxyapatite and gold nanoparticles were bound on the surface of the carbon nanotube, and dense particles were deposited on the wired surface. The gold nanoparticles were beneficial in forming the exceptional osteoimmune microenvironment to enhance cell multiplication. This structure facilitated the proliferation and attachment of bone cells. Sumathra et al. (Sumathra et al., 2020) developed the hydroxyapatite/κ-carrageenan−maleic anhydride/casein with doxorubicin (HAP/κ-CA-MA-CAS/DOX) composite. Then the composite was deposited on the titanium (Ti) plate. The DOX could induce cell regeneration and inhibition of cancer cells. The composite increased ALP’s activity, owning to DOX’s presence. The release system could provide enduring assistance in the self-repair and regeneration of bone affected by cancer cells due to the sustained release of the anticancer drug (DOX). Cells could not proliferate continuously because of the short half-life of growth factors. So, Jiang et al. (Jiang et al., 2018) use polycaprolactone (PCL) nanofibers and VEGF-encapsulated gelatin particles to prepare the sustained-release system. The system can release the growth factors stably to induce the differentiation of mesenchymal stem cells to endothelial cells.

The use of nanoparticles or microspheres in scaffolds has several advantages: i) nanoparticles or microspheres with small size, large specific surface area, and high porosity are more favorable to cell adhesion and proliferation and enormously improve loading rates of bioactive factor; ii) reduce bioactive factor degradation by enzymes and improve bone regeneration efficacy; iii) the strategy can load multiple types of bioactive factors simultaneously and regulate their release (Vo et al., 2012). In recent years, a growing number of scientists have focused on the indirect delivery systems in bone tissue engineering attributable to these evident benefits (Kim and Tabata, 2015; Wang et al., 2015; Li et al., 2020).

### 3.2 The releasing mechanism of bioactive factors

**FIGURE 3:**
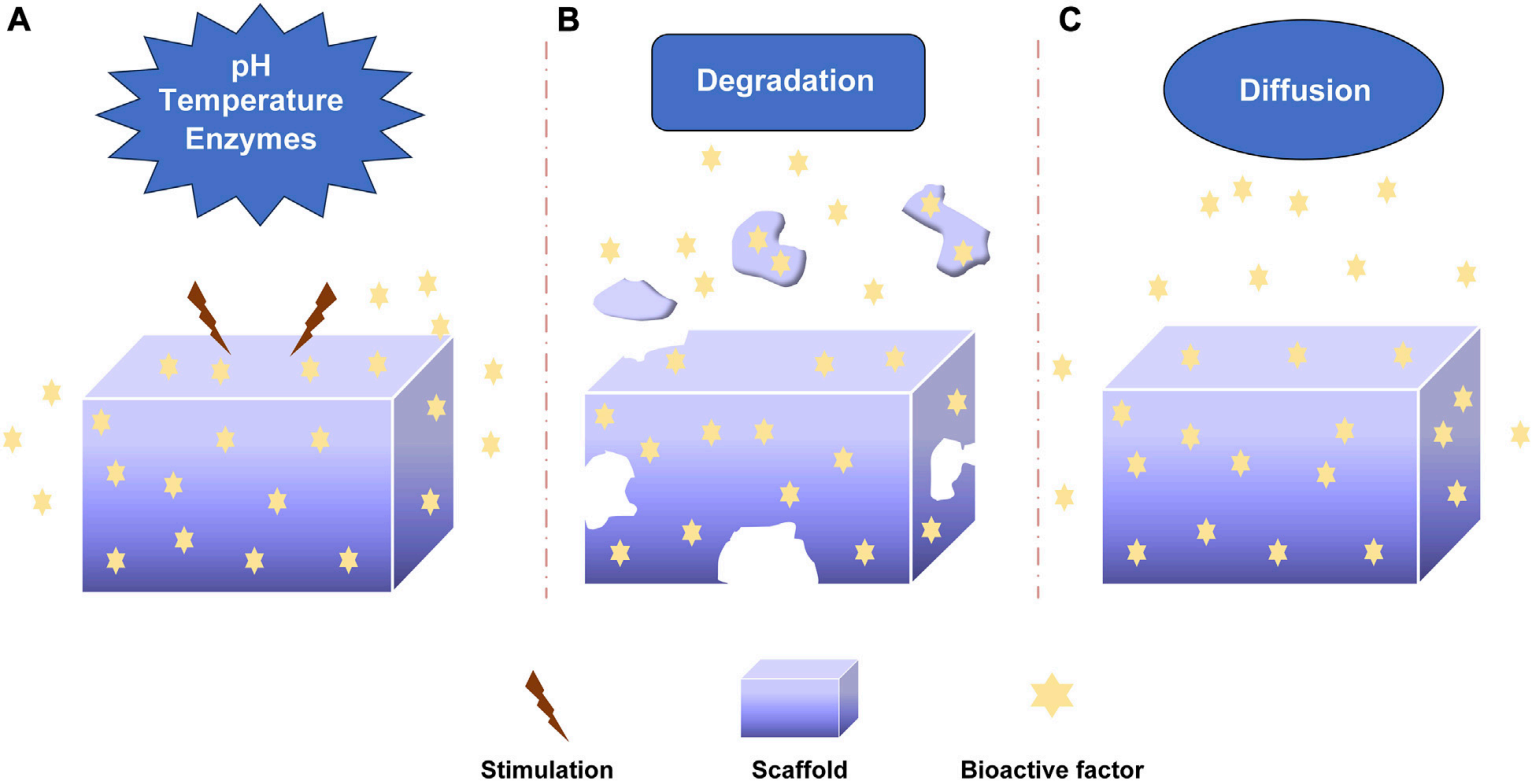
The releasing mechanism of bioactive factors. **(A)** Stimuli-responsive mechanism: pH, Temperature, Enzymes. **(B)** Degradation mechanism. **(C)** Diffusion mechanism.

The bioactive factors were loaded into scaffolds via non-covalent bonding, covalent bonding and particulate encapsulation, and they were released through diffusion, degradation, *etc.* In order to promote bone regeneration, understanding the release mechanisms of bioactive factors are essential for preparing and optimizing sustained-release systems. The release mechanism mainly included diffusion, degradation and stimulus responsiveness involving pH, temperature and enzymes (Figure 3). I would introduce these mechanisms in detail.

#### 3.2.1 pH-responsive mechanism

The pH-responsive mechanism was important in controlling the release of factors (Figure 3A). Different acidic or alkaline environments can influence the release of bioactive factors due to the different environments of organs, tissues, and cells (Shi et al., 2019; Lavanya et al., 2020; Zhang et al., 2021). This response was achieved by absorbing or releasing protons to change biomaterials’ deswelling, contraction, and other characteristics (Zhu and Chen, 2015). The weak polyelectrolyte poly (allylamine hydrochloride) can increase osmotic pressure when the pH decreases, leading to the diffusion of bioactive factors. The pH-responsive sustained release system was developed by Porta-i-Batalla et al. (Porta-i-Batalla et al., 2016) that tubular nanoporous anodic alumina membranes coated with polyelectrolytes. The burst release of DOX loading into scaffolds at pH 5.2 was faster than that at pH 7.4. The DOX released 90% within 24 h at PH 5.2, while the DOX released 30%–40% within 24 h at pH 7.4. Some sustained systems could alter the release rate of bioactive factors via PH variation. Matsusaki et al. organized the pH-responsive sustained release system with poly (γ-glutamic acid) (γ-PGA) and 72% sulfonated γ-PGA (γ-PGA-hetero-gels) loading fibroblast growth factor-2 (FGF-2). The system successfully controlled the release of (FGF-2) due to the sensitive deswelling properties at pH 2.0–6.0 (Matsusaki and Akashi, 2005).

#### 3.2.2 Temperature-responsive mechanism

Temperature-responsive sustained release system regulated the release rate by altering the temperature (Figure 3A). Thermo-responsive materials had reversible phase transitions with a specific temperature range. As an example, when the temperature rose to a specific temperature, deswelling properties changed for the thermally sensitive polymers to alter the release rate of bioactive factors (Dang et al., 2006; Izadifar et al., 2014). Wei et al. prepared thermosensitive micelles of a star block copolymer to control drug delivery. The copolymer comprised a hydrophobic PMMA arm and hydrophilic poly (N-isopropyl acrylamide) (PNIPAAm) arms. The PNIPAAm displayed a thermos-responsive phase transition at about 33°C. Based on this, the copolymer significantly enhanced drug release (Wei et al., 2007). The temperature-responsive drug delivery system was made by Zhu et al. with SBA-15/poly (N-isopropyl acrylamide) composite loading gentamicin. The gentamicin release experiment demonstrated the nature of temperature-responsive controlled release. (Zhu et al., 2009).

#### 3.2.3 Enzymes-responsive mechanism

The mechanism by which enzymes specifically respond to bioactive factors or target compounds is known as the enzyme-responsive mechanism (Figure 3A). The covalent binding-based controlled release systems often utilize hydrolysis or enzymolysis mechanism to regulate the release (Lavanya et al., 2020). Some biomaterials are enzyme-sensitive polymers that would alter the degradation of carrier materials to control the release of factors after adding enzymes. Some enzymes controlled the release by cleaving the bonds between the biomaterials and bioactive factors (Rezaei et al., 2022). Patel explored the controlled release of BMP-2 from gelatin *in vitro* and *in vivo*. The extent of gelatin crosslinking could influence the degradation of collagenase, and the addition of enzymatic increased the release rate of BMP-2. The release rate could be controlled by altering the extent of gelatin crosslinking. In addition, basic gelatin decreased the release rate. The enzymatic degradation and acid-base condition affected the sustained release (Patel et al., 2008). Phelps et al. developed polyethyleneglycol-based bioartificial hydrogel matrices. The presence of proteases promoted the degradation of scaffolds to release bioactive factors to facilitate vascular regeneration therapy (Phelps et al., 2010).

#### 3.2.4 Degradation mechanism

The degradation of carrier materials is another release mechanism of bioactive factors. The bioactive factors would be released when biological materials underwent hydrolysis or other types of degradation (Figure 3B). The degradation rate of biological materials influenced the sustained-release rate of bioactive factors (Nguyen and Alsberg, 2014; Rezaei et al., 2022). The researchers should select appropriate biomaterials for controlling the release. Krishnan et al. (Krishnan et al., 2020) prepared a fibrous scaffold (silica coated nanohydroxyapatite–gelatin reinforced with poly-L-lactic acid yarns) loading vancomycin for the treatment of osteomyelitis because of methicillin-resistant *Staphylococcus aureus* in rat models. The two loading strategies were done to investigate the effectiveness of treatment. That one was the vancomycin was loaded during the synthesis of the scaffold, and another was the vancomycin was added after the completion of a scaffold. The drug was released continuously by the degradation of the scaffold to encourage bone regeneration, and the effect of sustained-release was no difference between the two types of scaffolds. Verheyen et al. (Verheyen et al., 2010) developed a hydrogel scaffold and released VEGF via hydrogel degradation. The appropriate degradation of biomaterials could be able to maintain sustained release.

#### 3.2.5 Diffusion mechanism

The diffusion mechanism was involved in most sustained systems (Figure 3C). The bioactive factors were released from the porous structure of bioactive materials by diffusion from high concentration to low concentration (Davis and Leach, 2011; Ramburrun et al., 2014; Sabaghi et al., 2023). The diffusion rate was influenced by pore size, porosity, interconnectivity, *etc.* Whang et al. (Whang et al., 2000) investigated the impact of pore size on release kinetics with pore sizes ranging from 7 to 70 μm. The release mechanism of rhBMP-2 from dimensional scaffolds was diffusion, and the release rate was controlled by the pore sizes. Dexamethasone was encapsulated in hydrogels and released from hydrogels by diffusion. The release rate could be controlled by altering the crosslinking level (Kim et al., 2011). Berchane et al. (Berchane et al., 2007) prepared piroxicam-loaded PLG microspheres through emulsion technique. The research showed that the release rate was impacted by diffusion, polymer degradation and pore size. The diffusion mechanism was important to control the release rate of bioactive factors.

## 4 The effects of scaffold microstructure on slow-release system

The improved scaffold microstructure can optimize the release rates of bioactive factors to produce a sustained-release impact. For future scientific research, it is essential to comprehend the relationship between the release rate of bioactive factors and scaffold microstructure. The impacts of scaffold microstructure on slow-release systems are described below.

### 4.1 The effects of pore shape of scaffolds on the slow-release system

**FIGURE 4:**
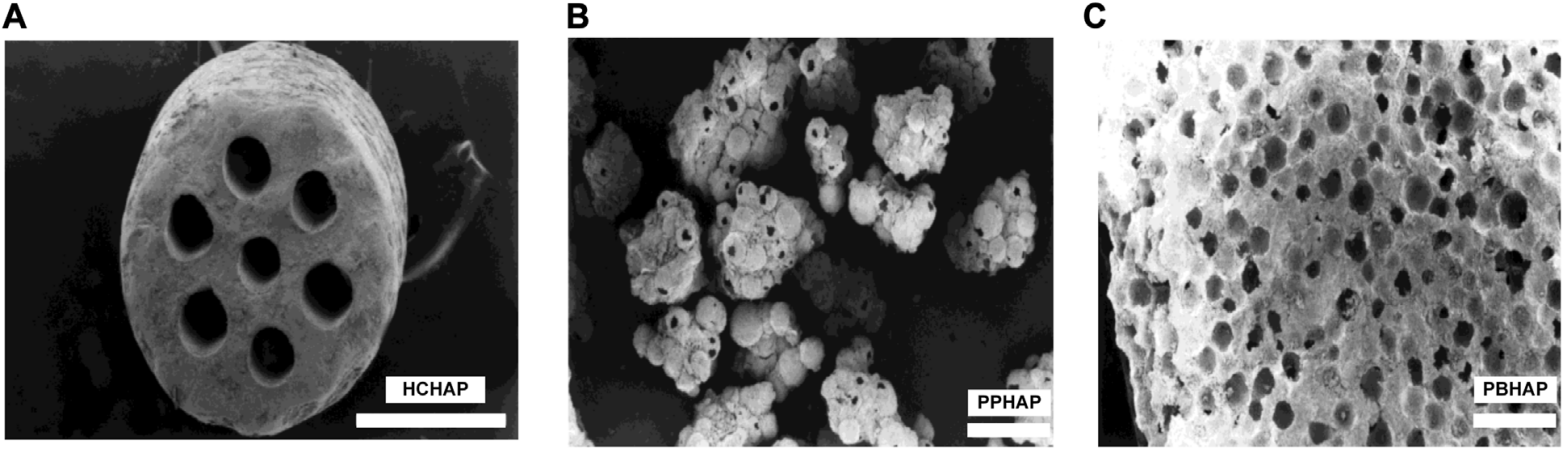
Three microscopic pore shapes of scaffolds. **(A)** HCHAP scaffold with 110 μm pore size. **(B)** PPHAP scaffold with average150 μm pore size. **(C)** PBHAP with average 150 μm pore size. Reproduced with permission from (Jin et al., 2000).

In the slow-release system, the pore shapes of scaffolds may impact the release of bioactive factors. In 1999, Jin et al. (Jin et al., 2000) produced three different pore-shaped hydroxyapatite systems with pore sizes of 100–200 μm to investigate the condition of bone formation, which were the honeycomb-shaped hydroxyapatite (HCHAP) system, the porous particles of hydroxyapatite (PPHAP) system and the porous blocks of hydroxyapatite (PBHAP) system (Figure 4).

**FIGURE 5:**
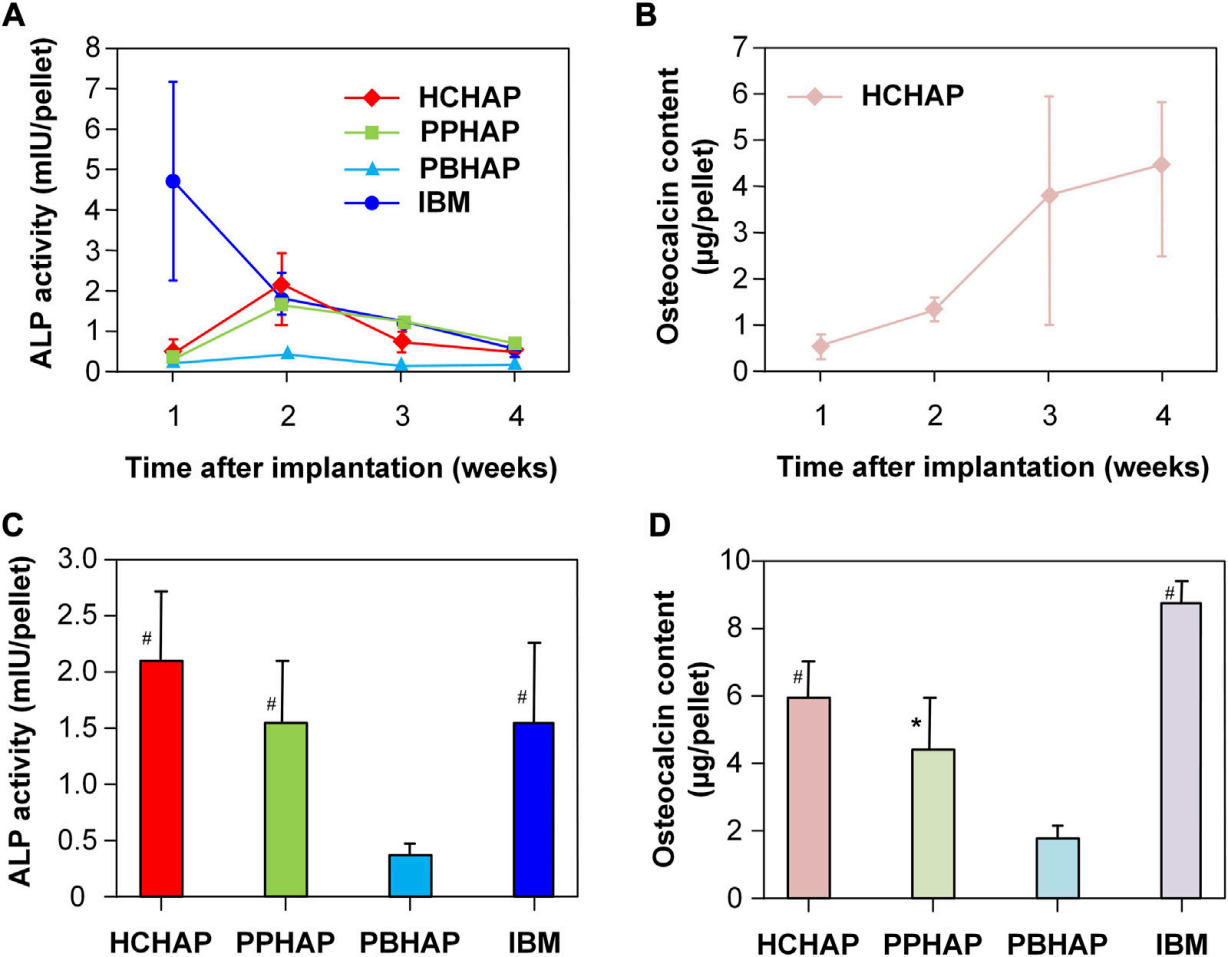
ALP activity and osteocalcin content in sustained systems. **(A)** Change of ALP activities in four samples in 4 weeks. **(B)** Change of osteocalcin contents in the PPHAP system in 4 weeks. **(C)** ALP activities in four samples at the second week. **(D)** Osteocalcin contents in four samples at the fourth week. (* indicates *p* < 0.05; # indicates *p* < 0.01). Reproduced with permission from (Jin et al., 2000).

BMP-2, a widely investigated growth factor, can increase alkaline phosphatase activity (ALP) and promote osteocalcin expression in a dose-dependent manner (Luvizuto et al., 2011; Kim et al., 2013; Li et al., 2015). Hence, the ALP activity and expression of osteocalcin can be used to evaluate the bioactivity and the slow-release effect of BMP-2. In biochemical analysis, the ALP activities and the osteocalcin content of all hydroxyapatite systems contain similar characteristics: i) ALP activities were increased from one to 2 weeks and decreased from three to 4 weeks, peaking in the second week (Figure 5A); ii) osteocalcin content was increased from one to 4 weeks, peaking in the fourth week (Figure 5B). The ALP activities of the HCHAP system were four times greater than those of the PBHAP system in the second week (Figure 5C), and the HCHAP system contained the highest osteocalcin content compared to the other systems (Figure 5D).

According to histologic observations, different types of bone formation can be seen. Simply put, the bone and cartilage were not formed in the first week, then discovered in the second week and continued to be formed in the PPHAP and PBHAP systems. In the HCHAP system, cartilage first developed in the tunnels and HCHAP particles, and in the second week, bone replaced the cartilage that had developed on the surfaces of the tunnels and HCHAP particles. The pore shapes influenced vascularization and oxygen diffusion, further affecting bone formation (Kuboki et al., 1995; Takita et al., 1997; Kuboki et al., 1998).

In the HCHAP system, the ALP activity and osteocalcin content were the highest. That meant the effect of rhBMP-2 on osteoinduction was better compared to those of other systems, and the bone formation depended on the pore shapes of the carriers. However, the difference in ALP activity and osteocalcin content between the HCHAP system and the PPHAP system was minimal, which led them to surmise that the pore shapes of carriers might have little impact on the release of bioactive factors.

Based on the literature evaluations, the pore shapes of scaffolds were not the only variable in recent studies. Table 2 illustrated that pore shapes of scaffolds might have little influence on the slow release of bioactive factors. This discovery resembled a previous study (Van Bael et al., 2012).

**TABLE 2:** Selected examples of slow-release systems possessing various pore shapes.

Pore shape	Carrier material	Bioactive factor	Porosity	Pore size	Release pattern	References
Fibrous structure	PCL-Gel-BCP	BMP-2	—	100nm-10 μm	32.1 %± 5.83%/1 day	Kim et al. (2014)
					54.4% ± 9.22%/7 days	
					83.16% ± 11.33%/31 days	
	PCL	BMP-6	—	377 ± 67 nm	12%/4 h	Toprak et al. (2021)
					35%/30 days	
Square open pore structure	MPHS	DMOG	—	—	40%/3 days	Min et al. (2015)
					90%/28 days	
Porous structure	PHB-pDA	BMP-2	86%	180 ± 10 μm	4.86% ± 3.2%/1 day	Li et al. (2019)
					95%/30 days	
	PHB				54.5% ± 9.6%/1 day	
					100%/16 days	
	HA	GM	40%	100–750 μm	25%/1 day	Mohan et al. (2018)
					50%/5 days	
					95%/20 days	
					100%/53 days	
	Mg-Zn	TCN	63%–65%	600–800 μm	30%–40%/6 h	Dayaghi et al. (2019)
					100%/16 h	
	DCB-ECM	TGF-β3	71%	67.76 ± 8.95 μm	40%/14 days	Yang et al. (2021)
					50%/42 days	

PCL-Gel-BCP: polycaprolactone-gelatin-biphasic calcium phosphate; MPHS: mesoporous bioactive glasses and poly (3-hydroxybutyrate-co-3-hydroxyhexanoate) polymers; DMOG: dimethyloxallyl glycine; PHB: poly (ε-caprolactone)-nanohydroxya-patite-bioglass; PHB-pDA: poly (ε-caprolactone)-nanohydroxya-patite-bioglass-polydopamine; HA: hydroxyapatite; GM: gentamicin; TCN: tetracycline; DCB-ECM: demineralized cancellous bone-acellular cartilage extracellular matrix.

For slow-release systems, the fibrous scaffolds can be obtained through electrostatic spinning, while porous scaffolds with round, triangular, and other pore shapes can be produced by 3D printing, heat treatment, and other methods. Although pore shapes may have no immense significance on the sustained release of bioactive factors, they do affect mechanical properties and cell differentiation (Moroni et al., 2006; Amirkhani et al., 2012; Boccaccio et al., 2016; Zhao D. et al., 2018; Zhang et al., 2020; Diez-Escudero et al., 2021). Zhao et al. (Zhao D. et al., 2018) fabricated porous titanium scaffolds by selective laser melting with different pore shapes (tetrahedron and octahedron) and pore sizes (500 μm and 1,000 μm). The octahedron scaffolds exhibited outstanding static mechanical properties, and the scaffolds with a pore size of 1,000 μm were more suitable for cell proliferation. Similarly, Boccaccio et al. (Boccaccio et al., 2016) investigated the effect of pore shapes on osteogenesis by numerical optimization methods and a computational mechano-regulation mode. The scaffolds with rectangular pores could produce more bone than those with square pores. Similarly, scaffolds with elliptic pores could form more bones compared to scaffolds with circular pores.

In this section, for slow-release systems, the pore shapes of scaffolds might bear limited significance in the release rates of bioactive factors. Still, they might influence mechanical properties, cell differentiation, bone formation, *etc.* Scaffolds with elliptical pores might be more conducive to bone formation, comparable to the pore shapes in human bones.

### 4.2 The effects of pore size of scaffolds on the slow-release system

Scaffolds with large pore sizes promote bone formation, and scaffolds with small pore sizes facilitate the transport of nutrients. The relationships between the release rates of bioactive factors and the pore sizes of scaffolds are shown in Table 3.

**TABLE 3:** Selected examples of slow-release systems possessing various pore shapes.

Carrier material	Bioactive factor	Pore size	Appropriate pore size for sustained release	References
Core (PEO)-shell (PCL-PEG) fiber mats	BMP-2	400, 450, 500, 650 nm	400 nm	Srouji et al. (2011)
HA	BMP-2	106–600 μm	300–400 μm	Tsuruga et al. (1997)
Cap	BMP-2	480, 720 μm (experiment)	480 μm	Sun et al. (2013)
		180–720 μm (simulation)	540 μm	
CSi-Mg6	Ion	480, 600, 720 μm	600 μm	Qin et al. (2022)
P (DLLA-co-TMC)	BMP-2	5.2 ± 0.9 μm	-	Wang et al. (2017)
		35.2 ± 13.9 μm		
PLLA	Protein	50–350 μm	Both 50–350 μm and 100 nm-10 μm	Wang et al. (2016)
		100 nm-10 μm both 50–350 μm and 100 nm-10 μm		

PEO: poly (ethylene oxide); PCL-PEG: polycaprolactone-poly (ethylene glycol); Cap: calcium phosphate; CSi-Mg6: 6 mol% Mg-substituted CSi (magnesium-substituted calcium silicate scaffolds; P (DLLA-co-TMC): poly (D, L-lactic acid-co-trimethylene carbonate); PLLA: poly (L-lactic acid).

In 1997, Eichi Tsuruga et al. (Tsuruga et al., 1997) investigated the effects of systems with different pore sizes on bone formation, which was the first demonstration that the pore sizes of carriers could influence cell differentiation. The researchers fabricated five porous hydroxyapatite scaffolds with pore sizes of 106–212, 212–300, 300–400, 400–500 and 500–600 μm with the same porosity (70%). They compared the ALP activities in the second week and osteocalcin contents in the fourth week with different pore sizes according to the biochemical analysis of implanted ceramics with a pore size of 106–212 μm. The ALP activities and the osteocalcin contents of the porous hydroxyapatite system with a pore size of 300–400 μm were 3.5 times and 2.0 times more than the system with a pore size of 106–212 μm, respectively.

Non-porous implants can neither develop cartilage nor osteogenesis, while small-porous implants can only form cartilage. Meanwhile, vascularization was the principal factor for BMP-induced ossification, which suggests the pores should be larger than the diameter of the capillary. Wang et al. (Wang et al., 2016) also demonstrated this point that the sizes of optimum macropores for neovascularization were 50–350 μm.

To sum up, it was considered that the implants with pore sizes of 300–400 μm were an applicable option that the slow-release systems had the highest ALP activities and osteocalcin contents analogous to some studies (Murphy et al., 2010; Murphy and O’Brien, 2010). The scaffolds with small pore sizes (90–150 μm) could promote cell and protein adhesion, whereas large pore sizes (>300 μm) could promote bone formation and blood vessel formation (Murphy et al., 2010; Murphy et al., 2010).

The point that the pore sizes of scaffolds impacted the release of bioactive factors could also be confirmed by simulation. Sun et al. (Sun et al., 2013) developed a 3D model to discover the effect of pore sizes on release rates of bioactive factors. They then carried out experiments to verify the simulation results. Experiments supported the simulation results that the release rate of bioactive factors in scaffolds with a small pore size (480 μm) was faster than in scaffolds with a large pore size (720 μm). The mechanism was explained by stating that more bioactive factors were required to be released into the environment in scaffolds with large pore sizes to reach a similar concentration as in scaffolds with small pore sizes. Like several other reports, the simulation also discovered that porosity had a stronger impact on angiogenesis and osteogenesis than pore size, which is more significant for releasing bioactive factors (Fisher et al., 2002; Karageorgiou and Laplan, 2005). In this study, the ideal pore size for releasing bioactive factors in the simulation model was 540 μm.

**FIGURE 6:**
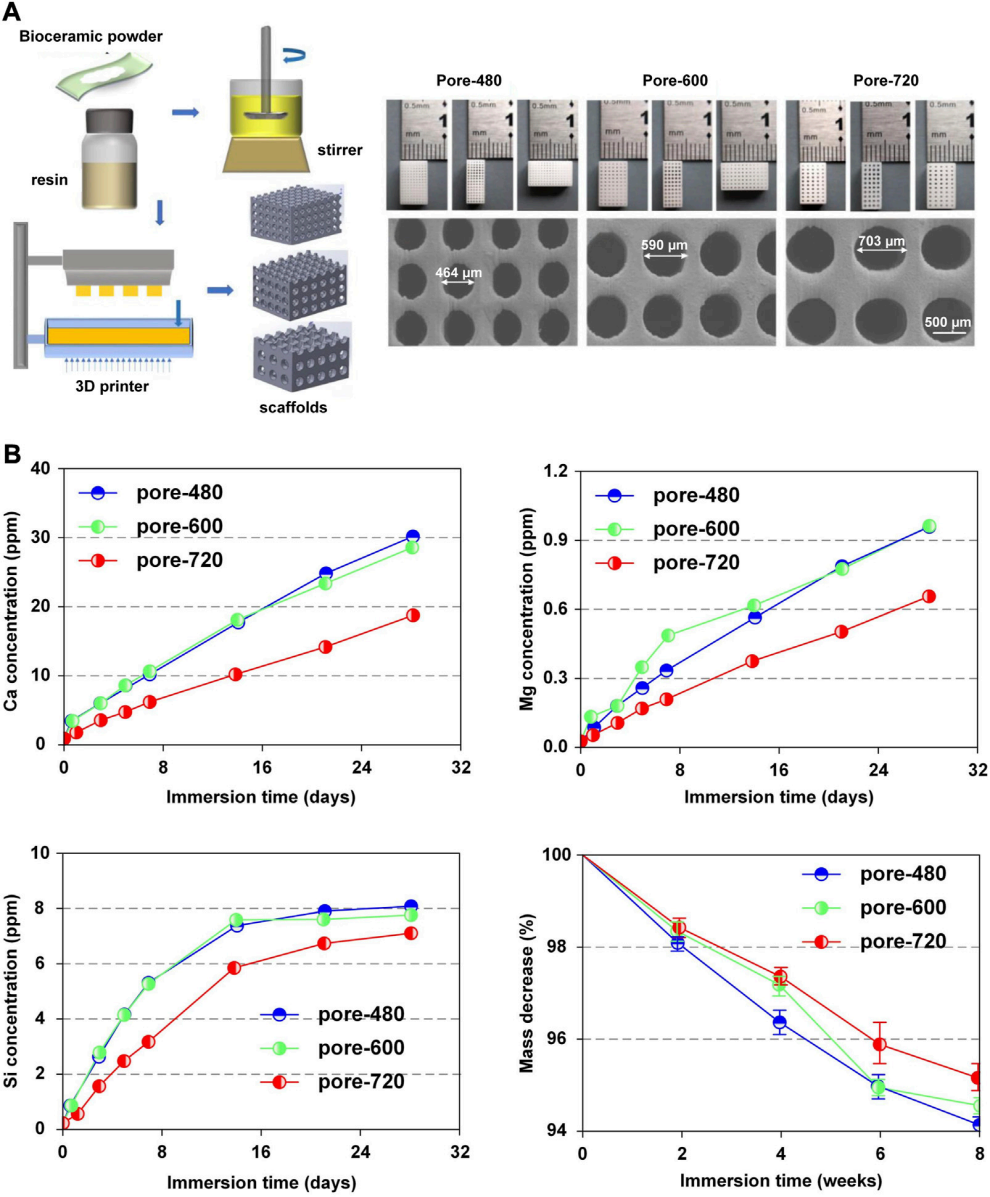
Release pattern of bioactive factors in slow-release systems. **(A)** Microscopic structures of CSi-Mg6 scaffolds with different pore sizes prepared by digital light processing-based three-dimensional printing technique. **(B)** Pattern of Ca, Mg, Si release, mass decrease in CSi-Mg6 scaffolds with different pore sizes. Reproduced with permission from (Qin et al., 2022).

Nevertheless, some researchers considered that scaffolds with larger pore sizes were more beneficial for slow release (Taniguchi et al., 2016; Szustakiewicz et al., 2021; Qin et al., 2022). Qin et al. (Qin et al., 2022) specialized in the system with 3D-printed bioceramic scaffolds with similar porosity but different pore sizes (480 µm、600 µm、720 µm) (Figure 6A). The ion release rate of the system with a pore size of 720 µm was lower than that of the other systems (Figure 6B). In this research, the scaffold with a pore size of 600 µm provided a satisfactory biological microenvironment in which the osteocytes had the best reproduction and bone was uniformly dispersed. Additionally, Taniguchi et al. (Taniguchi et al., 2016) did a series of experiments to support this point that the scaffold with a pore size of 600 µm was more suitable.

Some researchers investigated the effect of nanopores on slow-release systems. For instance, to investigate the bioactivity of growth factors, Samer et al. (Srouji et al., 2011) prepared core-shell fiber systems with different pore sizes using a co-electrospinning process.

The release of mats S1 and S2 with small pore sizes (400 ± 80 nm and 450 ± 77 nm) approached 12%–15% in 27 days, with a burst release of 5% in the first 4 h. And the release of mats S3 with a large pore size (500 ± 90 nm) approached 28% in 27 days, with a burst release of 18% in the first 4 h. While the release of mat S4 with a maximal pore size (650 ± 63 nm) approached 76% in 27 days and a burst release of 67% in the first 4 h. This experimental phenomenon demonstrated that the release speed with large nanopores was faster than that with small nanopores. The release pattern indicated that the release mechanism was related to desorption and was similar to the process described by Srikar et al. (Srikar et al., 2008). According to the diffusion equation (D_eff_ = D_bk_(t)/p_sp_) reported by Gandhi et al. (Gandhi et al., 2009), the diffusion speed increased with increasing pore size corresponding to the experimental result. Meanwhile, the slow-release systems had more significant ALP activity than fast-release systems, and the amount of new bone formation in slow BMP-2 releasing systems was 1.375 folds that of fast BMP-2 releasing systems.

**FIGURE 7:**
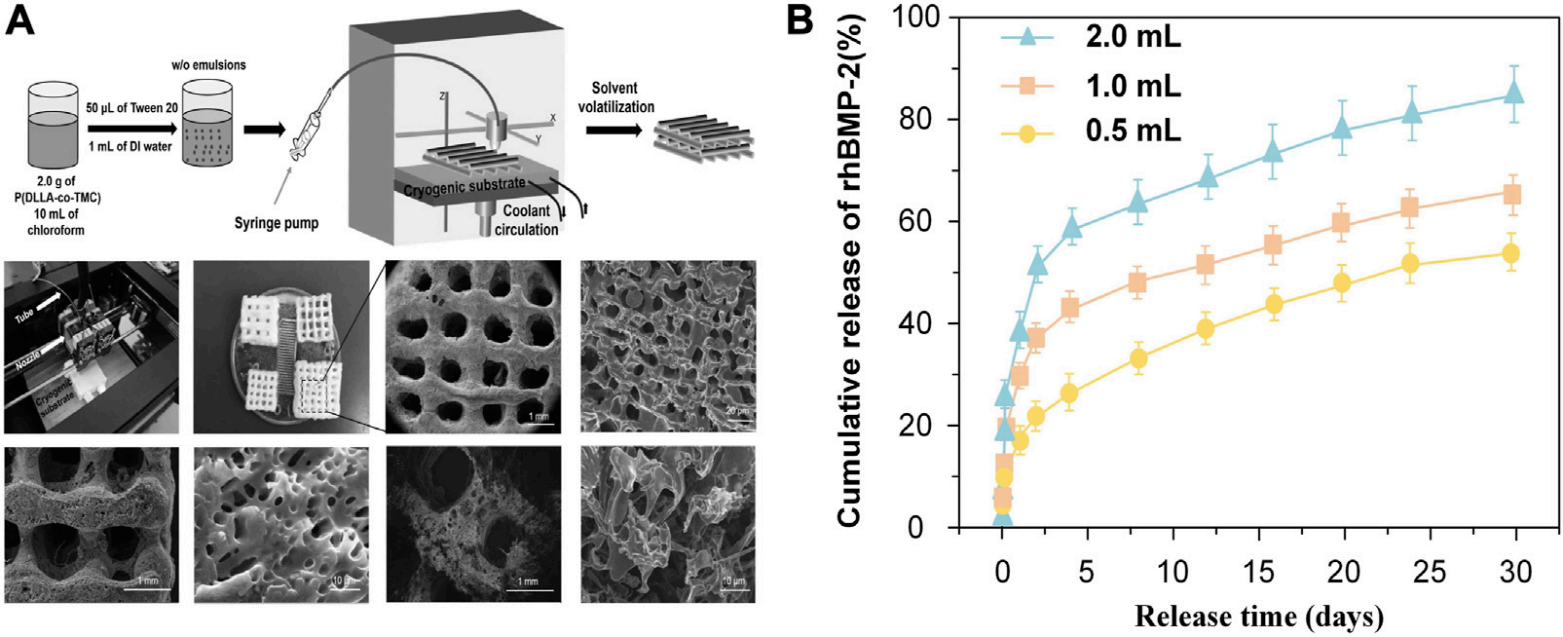
Release pattern of bioactive factors in slow-release systems with different pore sizes. **(A)** Schematic of P (DLLA-co-TMC) scaffold with different pore sizes. **(B)** Release pattern of BMP-2 in scaffolds with different pore sizes prepared with different water content emulsions. Reproduced with permission from (Wang et al., 2017).

Similarly, Wang et al. (Wang et al., 2017) also demonstrated that the diffusion speed expanded with the increase of pore sizes. They fabricated surface porous tissue engineering scaffolds through cryogenic 3D plotting (Figure 7A). The scaffold micropores changed from a diameter of 5.2 ± 0.9 µm to 35.2 ± 13.9 µm while a burst release of BMP-2 changed from 17% to 40% in the initial 24 h, and a sustained release of BMP-2 varied from 53% to 84% within 30 days (Figure 7B).

In slow-release systems, the macropores could stimulate the formation of blood vessels and cell proliferation and migration, while micropores could promote the release of nutrients. The slow-release systems with simplex pore size impact the release rate of factors and ALP activity. Slow-release systems containing macropores and micropores might be more appropriate. Wang et al. (Wang et al., 2016) developed four PLLA slow-release systems, including macropores (50–350 µm) and micropores (100nm-10 µm). The slow-release system with macropores and micropores provided a more favorable microenvironment for protein adsorption and cell proliferation than the other three slow-release systems with single-pore scaffolds.

The release rates of bioactive factors might increase with the increase of pore sizes in scaffolds with small pore sizes (nano-sized pores to tens of microns pores) and might decrease with the addition of pore sizes in scaffolds with large pore sizes (hundreds of microns pores). The reason could be that a sufficiently high concentration of bioactive factors was required to fill the pores first in scaffolds with larger pore sizes. Through investigation, it was discovered that the slow-release system with a large pore size (about 400 μm) might be more conducive to the slow release of bioactive factors to form bone. It seemed more acceptable to have both macropores and micropores in the scaffolds for a slow-release system.

### 4.3 The effects of porosity of scaffolds on slow-release system

The porosity of human cancellous bone ranges from 50% to 90%, implying that porosity beyond 50% is the essential element for cell proliferation and osteogenesis in bone tissue engineering (Batool et al., 2021; Oliveira et al., 2021). The following formula calculates porosity:

$$P=\frac{V_{0}-V_{2}}{V_{1}-V_{0}}\times 100$$ (4.1)

Where (p), (V0), (V1) and (V2) represent porosity (%), the initial volume of solvent (mL), the volume of solvent after the scaffold immersed (mL) and the volume of solvent after the scaffold removed (mL), respectively.

The porosity of scaffolds was generally above 70% and most were between 70% and 80%, with which the slow-release systems could regulate the release rates of bioactive factors to achieve the slow-release effect, as shown in Table 4.

**TABLE 4:** Selected examples of slow-release systems possessing various porosity.

Porosity	Pore diaeter	Carrier material	Bioactive factors	Release pattern	References
86.0%	180 ± 10 μm	PHB-pDA	BMP-2	4.86% ± 3.2%/1 day	Li et al. (2019)
				95%/30 days	
63.0%–65.0%	600–800 μm	Mg-Zn	TCN	30%–40%/6 h	Dayaghi et al. (2019)
				100%/16 h	
71.0%	67.76 ± 8.95 μm	DCB-ECM	TGF-β3	40%/14 days	Yang et al. (2021)
				50%/42 days	
69.4%	480 μm	CSi-Mg6	Ion	Fast release for 480 μm slow release for 720 μm	Qin et al. (2022)
69.8%	600 μm				
62.9%	720 μm				
73.6%	431.31 ± 18.40 μm	PLGA-n HA	BMP-2	9.54% ± 0.86%/2 days	Deng et al. (2019)
				61.38% ± 2.39%/30 days	
64.6%	125 μm	CS-HA	Icariin	50%/8 h	Li et al. (2013)
				73%/24 h	
				80%/2 days	
82.3%	400 μm	n BG-PS-COL	Steroidal saponin	18%/2 days	Yang et al. (2017)
				60%–70%/15 days	
				80%/35 days	
80.0%	400–600 μm	MBG-SA-G	Naringin and calcitonin gene-related peptides	Sustained release for 21 days	Wu et al. (2019)

PLGA-nHA: polylactic-coglycolic acid-nano-hydroxyapatite; CS-HA: chitosan-hydroxyapatite; nBG-PS-COL: nano-bioglass-phosphatidylserine-collagen; MBG-SA-G: mesoporous bioactive glass-sodium alginate-gelatin.

**FIGURE 8:**
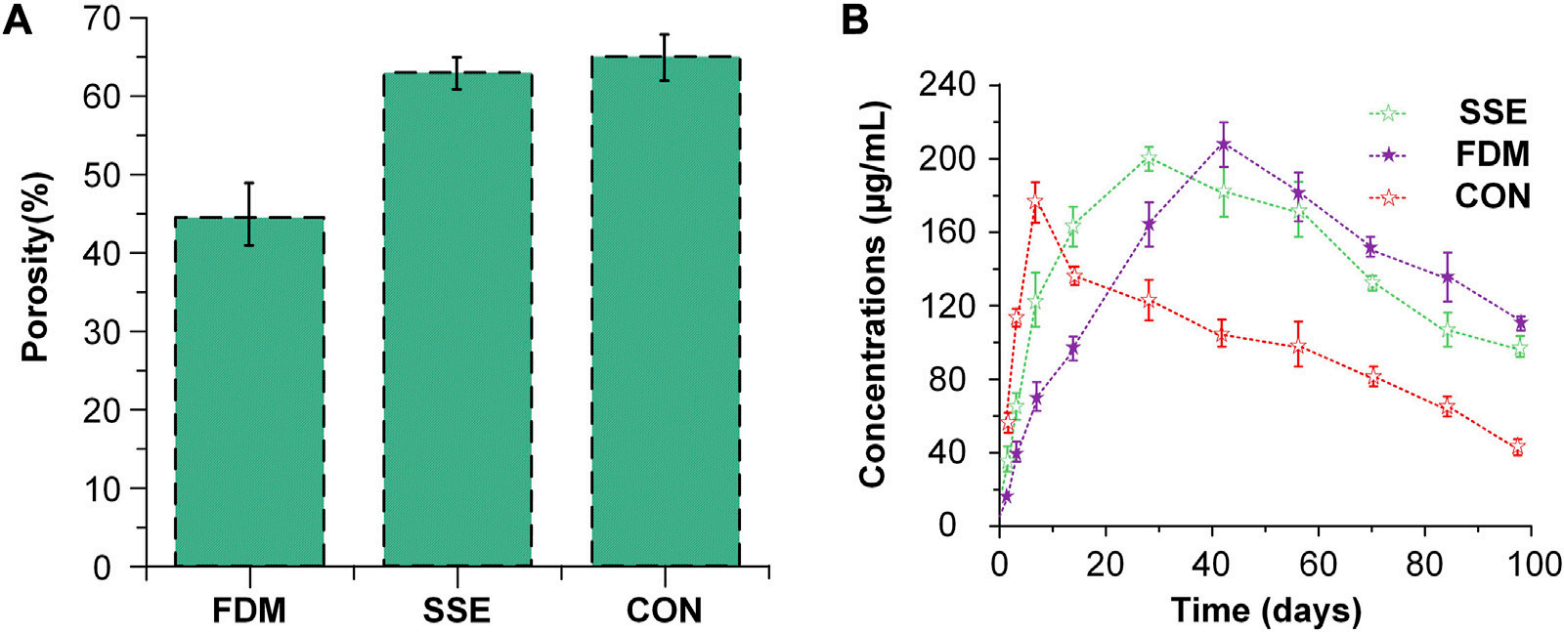
The situation of porosity and drug release of three slow-release systems **(A)** The porosity of the three scaffolds fabricated via three methods. **(B)** Drug release pattern in scaffolds with three different porosities. Reproduced with permission from (Cui et al., 2021).

The porosity and release rates might have a favorable correlation. Cui et al. (Cui et al., 2021) produced three types of slow-release systems with different porosities through three different techniques SSE 3D printing, FDM 3D printing, and a traditional approach (CON). The porosity of the scaffold prepared by CON was the largest at 64.96%. The drug release rate was also the fastest (Figure 8) compared to the other methods, and the mechanical properties and pore sizes also influenced the release. This discovery was similar to the research investigated by Qin et al. (Qin et al., 2022).

Similarly, Cheng et al. fabricated Ti-6Al-4V scaffolds by laser sintering with porosity varying from 15% to 70%. They discovered that when the porosity grew, the total amount of bioactive factors also did so, and the effect on osteogenesis was best in the system with the highest porosity (Cheng et al., 2014). In addition, the scaffolds with larger pore sizes might have larger porosities (Fu et al., 2010; Sobral et al., 2011; Liu et al., 2013; Cheng et al., 2014; Diao et al., 2018; Zaharin et al., 2018; Lee et al., 2019; Wo et al., 2020). But it does not mean the phenomenon is typical. For example, the discrepancy in pore size is apparent, but the difference in porosity is minimal (Al-Munajjed et al., 2008; Park et al., 2019). Amir A et al. (Al-Munajjed et al., 2008) fabricated hyaluronan-collagen scaffolds with three different pore sizes (302.5, 402.5 and 525.0 μm) but similar porosities (94.0%, 94.0% and 95.0%). The slow-release systems can have the same porosities but different pore sizes by 3D printing and other methods (Wo et al., 2020).

For efficient sustained release, the porosity was typically between 70% and 80%, and the porosity was independent of the pore size. Additionally, we observed that the release rate and amount of bioactive factors might increase when porosity increased.

### 4.4 The effects of other aspects on slow-release system

The release rates of bioactive factors could be impacted by a variety of aspects, not just the scaffold microstructure, including the common action of diverse bioactive factors, the encapsulation methods, pore interconnectivity and external factors (Hu et al., 2014; Rahman et al., 2014; Porta-i-Batalla et al., 2016; Bose et al., 2019; Kim et al., 2020; He et al., 2021; Hu et al., 2021).

The interconnectivity of pores plays a vital role in cell growth, migration, transport of nutrients and release of bioactive factors. There is a specific correlation between porosity and pore interconnectivity. In general, under the premise of satisfying mechanical properties, higher porosity is conducive to improving the interconnectivity of pores. The higher porosity and interconnectivity of pores benefit cell migration, transport of nutrients, and release of bioactive factors to stimulate bone regeneration. Wenk et al. (Wenk et al., 2009) prepared the silk fibroin scaffolds loading the insulin-like growth factor I, and the scaffolds possessed the nature of interconnectivity of pores. The method can increase the interconnectivity of pores to control the release of factors, in which the porogen was eluted with hexane and silk fibroin was transformed into water-insoluble conformation via methanol or water vapor. The porous polyethylene glycol diacrylate hydrogel system loading vascular endothelial growth factor (VEGF) was prepared. The performance of highly interconnected pores and favorable porosity is enough to release VEGF and promote angiogenesis (Oliviero et al., 2012). Kundu et al. (Kundu et al., 2011) organized antibiotic-loaded bioactive glass porous scaffolds to treat osteomyelitis. The vacuum infiltration and freeze-drying methods were used to load the drug onto scaffolds. The scaffold with 60%–65% porosity, 60 µm pore size, and high interconnectivity was ideal. The system with 49% antibiotic adsorption positively impacted bone formation. The efficient interconnectivity could promote the transport of nutrients and the release of bioactive factors to achieve the purpose of bone regeneration.

Some researchers incorporated one bioactive factor into microspheres and combined them with biomaterials to form scaffolds to achieve the slow-release effect (Fei et al., 2008; Chen et al., 2010; De Witte et al., 2018). Fei et al. (Fei et al., 2008) researched a novel composite slow-release system of bone grafts made of calcium phosphate cement (CPC) and PLGA microspheres that are loaded with rhBMP-2. Compared to the CPC system, the composite system properly extended the release of rhBMP-2 such that the composite system had an initial release of 4.9% in 24 h and a prolonged release for 28 days. Some researchers have encapsulated two bioactive factors into a slow-release system to release bioactive factors sequentially to achieve the slow-release effect (Schmidmaier et al., 2003; Raiche and Puleo, 2004; Yilgor et al., 2009; Lim et al., 2010). Lim et al. (Lim et al., 2010) developed a delivery system for BMP-7 and TGF-β2 where BMP-7 and TGF-β2 were loaded into gelation alginate and polyion complex nanoparticles, respectively. The release rates of dual growth factors (BMP-7: 36% and TGF-β2: 16%) in the nanoparticle/hydrogel system were much slower than the respective release rates (BMP-7: 70% and TGF-β2: 50%). External factors were also vital for the release rates (Hu et al., 2014; Porta-i-Batalla et al., 2016; Kim et al., 2020; Park et al., 2021). Porta et al. examined the different release models of doxorubicin at PH 5.2 and 7.4 with a faster release rate at PH 5.2. The various situations emphasize the significance of comprehensive consideration for a suitable slow-release system.

## 5 Conclusion and future prospects

Slow-release systems improve the limitations of bioactive factors to promote further bone repair, which develops the application of BTE. Hence, slow-release systems play an irreplaceable role in BTE. This review first summarizes the requirements and preparation methods of ideal scaffolds, followed by some characteristics of scaffold microstructure. Subsequently, the loading nature and releasing mechanism of bioactive factors were described. The bioactive factors were incorporated into scaffolds with non-covalent or covalent bonding. In addition, in the third delivery strategy, the bioactive factors were encapsulated into nanoparticles or microspheres and loaded into scaffolds. The releasing mechanism contained diffusion, degradation and stimulus responsiveness involving pH, temperature and enzymes. Finally, this review primarily epitomized the impacts of scaffold microstructure, including pore shape, pore size and porosity on slow-release systems with bioactive factors for bone repair.

The pore shapes of scaffolds might have minimal influence on the release rates of bioactive factors but significantly influence the mechanical properties of slow-release systems and bone differentiation. Previous studies have shown that pores are the basic requirement for producing bone (Tsuruga et al., 1997) that elliptic pores have an enormous potential for osteogenesis (Boccaccio et al., 2016). Blood vessels could develop when the pore sizes were larger than 50 um, and the slow-release systems with large pore sizes (about 400 µm) might have more potential to control the release rates of bioactive factors. The regular distribution of pore sizes from nanometer to micron is more conducive to bone regeneration. In terms of porosity, the porosity of scaffolds was generally beyond 70%, and most were between 70% and 80%. When the porosity is relatively high, the release rates of bioactive factors might be relatively fast. Furthermore, there is no strong correlation between the porosity and the pore size in the slow-release systems.

We realized that it was challenging to prepare a suitable sustained-release system. The bioactive factors loaded into scaffolds with non-covalent bonding, especially physical adsorption, would have burst release in the preliminary period. This type of system had the property of burst release in the initial stage and slow release in the later stage. It is not conducive to the stable release of bioactive factors and the regeneration of bone tissue. The bioactive factors loaded into scaffolds with covalent bonding, such as primary amine, carboxyl, *etc.*, would be more effective in reducing burst release in the preliminary period and maintaining sustained release in the whole process. This kind of system could better sustain the release of bioactive factors and promote bone tissue regeneration. Also, the bioactive factors loaded into scaffolds with particulate encapsulation could control the release of bioactive factors. The nanoparticles or microspheres with small sizes and large specific surface areas could be more advantageous for releasing factors. We realized that the system with about 400 µm pore size and about 70%–80% porosity might be more potential to control the release rates of bioactive factors. We considered that the system in which bioactive factors were loaded into scaffolds covalently with about 400 µm pore sizes with 70%–80% porosity and the system in which bioactive factors were encapsulated into particles and then loaded into scaffolds could be more possible to retain long-term steady release of bioactive factors. But the release rate could also be influenced by many other elements, including pH, temperature, the concentration of bioactive factors, *etc.* We should consider various aspects more deeply to get a relatively perfect sustained system.

Several issues still need to be resolved in the future, although the slow-release systems have significantly advanced bone regeneration. The first challenge is related to bioactive factors in slow-release systems. Due to the complexity of the process, various bioactive factors with different efficacies are involved in bone regeneration. But the research on the slow-release systems involving multiple bioactive factors to simulate the natural healing cascade is limited to a limited number of studies, which needs to be further performed. Another is the translation of scientific research into clinical applications. Most research experiments have been conducted on animals such as mice or rabbits, but their applicability to the human body needs to be clarified. I suggest that scientists conduct human osteoblast experiments on amphibians to expand the potential for clinical applications. Slow-release systems have brought us great inspiration in the treatment of bone diseases. More and more scientific research on slow-release systems will be transformed into clinical applications to address therapeutic issues excellently in the future.
